# Passive Root Coverage Through Creeping Attachment of the Gingiva Before and After Scaling and Root Planning Therapy in the Mandibular Anterior Teeth: A Clinical Study

**DOI:** 10.7759/cureus.57734

**Published:** 2024-04-06

**Authors:** Annapurna Ahuja, Vipin Ahuja, Rakhi Kumari, Nilima R Thosar

**Affiliations:** 1 Periodontics and Implant Dentistry, Hazaribag College of Dental Sciences and Hospital, Hazaribag, IND; 2 Pediatric and Preventive Dentistry, Hazaribag College of Dental Sciences and Hospital, Hazaribag, IND; 3 Pediatric and Preventive Dentistry, Sharad Pawar Dental College and Hospital, Datta Meghe Institute of Higher Education and Research (Deemed to Be University), Wardha, IND

**Keywords:** scaling and root planning, phase i periodontal therapy, passive root coverage, creeping attachment, gingival margin

## Abstract

Introduction

Root coverage is one of the most imperative procedures in periodontal therapy. The demands from patients for aesthetics and sensitivity are some of the complaints in root exposure cases. Creeping attachment is a passive apical migration of the marginal gingiva and can be proposed as a noteworthy root coverage technique. The purpose of this study was to evaluate the position of the marginal gingiva and quantify the creeping attachment before and after the scaling and root planning (SRP) procedure.

Materials and methods

The present study was based on a single-centre clinical trial in which 30 sites from 10 patients were selected. Patients with Miller’s class I gingival recession were selected. The plaque index, gingival biotype, and gingival margin position were measured at baseline and then at 10 and 21 days after SRP. The gingival biotype was measured with an endodontic file with a stopper and a digital vernier calliper. The gingival margin position was measured from the incisal surface of the tooth to the marginal gingiva with the help of a University of North Carolina (UNC) 15 periodontal probe. After recording the clinical parameters, a thorough SRP was performed using an ultrasonic scaler and manual instruments.

Results

The results were compared clinically at baseline and after 10 days and 21 days postoperatively. Statistical analysis was conducted for pre-SRP and post-SRP findings using the IBM SPSS Statistics for Windows, Version 19 (Released 2010; IBM Corp., Armonk, New York) software. A statistically significant improvement was seen in all the clinical parameters at baseline, at 10 days, and at 21 days postoperatively after the procedure (P < 0.01).

Conclusion

The position of the gingival margin was shifted towards the crown, and the creeping attachment was significantly observed from baseline to 10 and 21 days.

Clinical significance

Creeping attachment after the SRP procedure can be considered a significant root coverage technique. The genetic memory of the gingiva may play an important role in achieving root coverage. Once local irritants are removed, the gingiva attempts to regain its original position. In addition, the gingival phenotype plays an important role in gingival marginal positioning after basic therapy and root coverage procedures.

## Introduction

Gingival recession is an aesthetic issue, distressing nearly all individuals to some degree. It involves the apical relocation of the gingival margin, uncovering the root surface. The space between the cementoenamel junction (CEJ) and the gingival margin indicates the extent of recession. Gingival recession can be caused by many factors, such as faulty tooth brushing, poor oral hygiene, calculus accumulation, improper flossing, incorrect occlusal relationships, mispositioning of a tooth out of the arch, and faulty restorations. It causes not only aesthetic problems but may also lead to root hypersensitivity because of the exposed dentin. Various root coverage procedures are available to treat the exposed root surfaces, such as masking the exposed roots using gingival veneers or surgically correcting the gingival position to cover the denuded root surface. When considering these options, it is important to take note of the genotype memory of periodontal tissues, whereby gingival tissue tries to return to its former healthy state. This process is termed creeping attachment. The term is not new in the literature. Creeping attachment of the gingiva was first described by Goldman and Cohen in 1964 in relation to passive root coverage post-treatment after healing. It is defined as the post-operative movement of the marginal tissues of the gingiva towards the crown route along areas of formerly denuded root [1,2].

The relocation of the gingival margin towards the crown does not appear to occur at a perpetual rate but rather as the consequence of consecutive incidents of recession and creeping. Rationally, this process can be explained by the resolute arrangement and maturation of connective tissue; creeping attachment is one of a variety of treatment modalities used for coverage of the denuded root. Documented studies have an inadequate understanding of the time and the point when creeping attachment starts and halts, as well as when to assess this procedure. The mechanism of creeping attachment formation is always seen with an eye of speculation. There are mainly case reports wherein passive attachment is frequently observed after mucogingival surgeries [3-6]. Orthodontic correction also leads to the formation of creeping attachment on exposed root surfaces. There is a plethora of literature to prove that orthodontic therapy, along with grafts, initiates this procedure. On the contrary, very few case reports inferred that this attachment formed due to orthodontic corrections and not the gingival grafting procedure. One of the influencing factors in the formation of creeping attachment is the eradication of bacterial load and toxins from the site; hence, scaling and root planning (SRP) in phase I periodontal therapy may promote its formation. It is also documented that creeping attachment is primarily observed in the mandibular anterior teeth. Gingival self-healing can be seen as a kind of attempt to restore dynamic equilibrium within the body, whereby the gingiva tries to heal after the removal of irritants and tries to shift to its original position before its apical migration due to loss of attachment [7]. Considering all these factors, we conducted this research to determine whether creeping attachment can be initiated and formed after the SRP procedure alone. To date, no study has thoroughly explored the effects of basic periodontal treatment on gingival margin location. Consequently, this research was intended to understand the effects of phase I periodontal treatment on the position of the marginal gingiva and passive root coverage in the mandibular anterior teeth.

## Materials and methods

Study area

A single-centre trial was conducted from December 2022 to April 2023. Institutional ethical board approval was obtained prior to the commencement of the study. Ten patients, aged between 25 and 55, were recruited from the outpatient section of the Department of Periodontology and Oral Implantology, Hazaribag College of Dental Sciences and Hospital, Hazaribag, India. Patients were selected based on Miller’s gingival recession classification [8]. Before recruiting participants, informed consent was obtained. Patients were briefed about the procedural steps of the study.

Sample size calculation

The sample size calculation was conducted using the G*Power 3.1.9.4 software. The effect size was maintained at 0.6, the alpha error was set at 5%, and the power of the study was 80%. The study requires a total sample size of 30 units (sites). So, we have taken this sample size of 30 sites from 10 subjects.

Ethical approval

The ethical clearance was obtained from the Institutional Ethics Committee of Hazaribag College of Dental Sciences and Hospital, Hazaribag, India (HCDSH/IEC/CERT/2023-24/162).

Inclusion and exclusion criteria

Patients with Miller’s class I gingival recession were selected. In these cases, marginal gingiva well above the mucogingival junction without any soft or hard tissue loss interdentally was requisite. Patients with mobile teeth, trauma from occlusion, abnormal frenum attachment and systemic diseases, as well as lactating mothers, pregnant ladies, and smokers, were excluded.

Data collection

**Figure 1:**
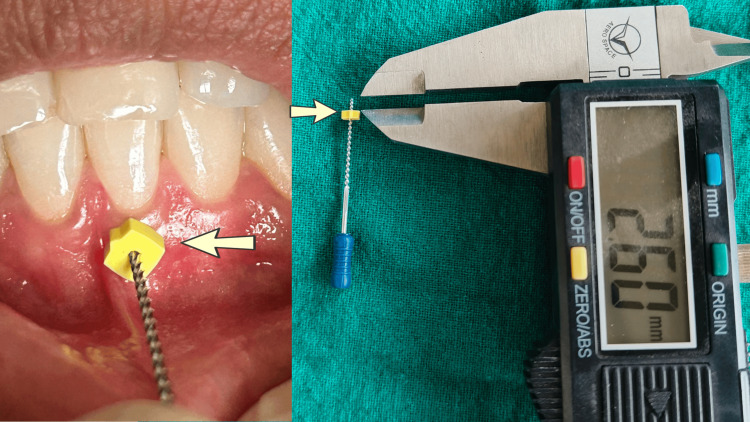
Recording of gingival thickness with an endodontic file and its measurement using a vernier calliper

The plaque index (Silness and Loe), gingival biotype, and gingival margin position (gingival recession) were measured at baseline and then at 10 and 21 days after the SRP procedure. The gingival biotype was measured with an endodontic file with a stopper and a digital vernier calliper. A topical anaesthetic gel was applied to the attached gingiva, and the reamer was pressed onto the attached gingiva until it reached the bone surface. At this point, the stopper was adjusted, and readings were taken using the vernier calliper. The recording of gingival thickness using an endodontic file, followed by its measurement using a vernier calliper, is shown in Figure 1.

The gingival margin position was measured from the incisal surface of the tooth (with gingival recession) to the marginal gingiva with the help of a University of North Carolina (UNC) 15 periodontal probe. The SRP procedure was done using ultrasonic scalers and manual curette instruments. Oral hygiene instructions were given, including gingival massaging in a circular motion to improve blood circulation and enhance healing. Patients were recalled for re-evaluation after 10 days and after 21 days.

Recall protocol

In the present study, an attempt has been made to evaluate the effects of phase I periodontal treatment on the position of marginal gingiva and passive root coverage in the mandibular anterior teeth. This clinical study included 10 patients and 30 sites observed for three weeks after the SRP procedure. The recall protocol of three weeks adheres to the guidelines proposed by Chilton and Fleiss for conducting trials on gingival inflammation with a study period exceeding two weeks [9].

## Results

The aim of this study was to evaluate the efficacy of basic periodontal therapy on the gingival margin position and passive root coverage. A total of 30 sites from 10 subjects were analyzed before and after SRP. A comparison was made between the gingival margin positions and creeping attachment, measured in millimetres (mm) at the pre-SRP stage and post-SRP after 10 and 21 days (Table 1). The results are shown following an analysis of variance (ANOVA) test. A statistically significant improvement was observed from baseline to 10 and 21 days, with values of 10.2 ± 1.13, 8.4 ± 1.71, and 8.2 ± 1.54, respectively, with a P-value of 0.01.

**Table 1:** Comparison of creeping attachment at different time intervals before and after scaling and root planning (SRP)

Study groups	Creeping attachment (mean ± SD)	F-value	P-value
Group A (Pre-SRP)	10.2 ± 1.13	5.49	0.01 (P < 0.05), Significant
Group B (Post-SRP, after 10 days)	8.4 ± 1.71		
Group C (Post-SRP, after 21 days)	8.2 ± 1.54		

A comparison of gingival thickness in millimetres (mm) at the pre-SRP stage and post-SRP after 10 and 21 days using the ANOVA test is shown in Table 2. The values are 2.75 ± 0.67, 2.5 ± 0.47, and 2.4 ± 0.51, respectively, with a P-value of 0.37, indicating that it is not statistically significant.

**Table 2:** Comparison of gingival thickness at various time intervals before and after scaling and root planning (SRP)

Study groups	Gingival thickness (mean ± SD)	F-value	P-value
Group A (Pre-SRP)	2.75 ± 0.67	1.02	0.37 (P > 0.05), Not significant
Group B (Post-SRP, after 10 days)	2.5 ± 0.47		
Group C (Post-SRP, after 21 days)	2.4 ± 0.51		

A comparison of plaque index scores at different time intervals is shown in Table 3. The values were 1.58 ± 0.33, 0.61 ± 0.09, and 0.54 ± 0.10 at baseline, 10 days, and 21 days, respectively.

**Table 3:** Comparison of plaque index score at various time intervals before and after scaling and root planning (SRP)

Study groups	Plaque index score (Silness and Loe) (mean ± SD)	F-value	P-value
Group A (Pre-SRP)	1.58 ± 0.33	76.3	0.0000 (P < 0.001), Highly significant
Group B (Post-SRP, after 10 days)	0.61 ± 0.09		
Group C (Post-SRP, after 21 days)	0.54 ± 0.10		

**Figure 2:**
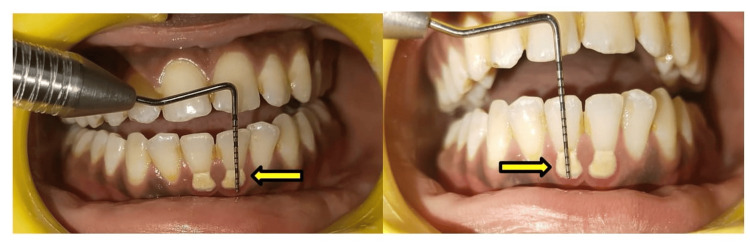
Pre-scaling and root planning (SRP) measurement of margin position at teeth nos. 31 and 41

**Figure 3:**
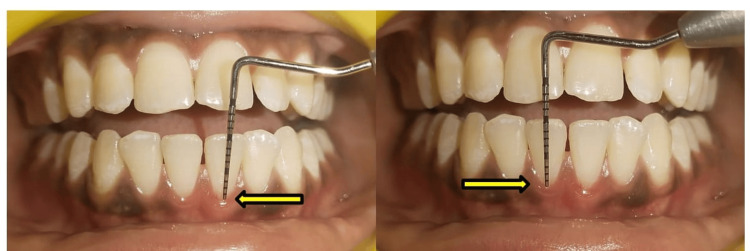
Post-scaling and root planning (SRP) measurement of margin position at teeth nos. 31 and 41 after 21 days

The gingival margins of teeth nos. 31 and 41 are measured at the pre- and post-SRP stages after 21 days (Figures 2, 3).

**Figure 4:**
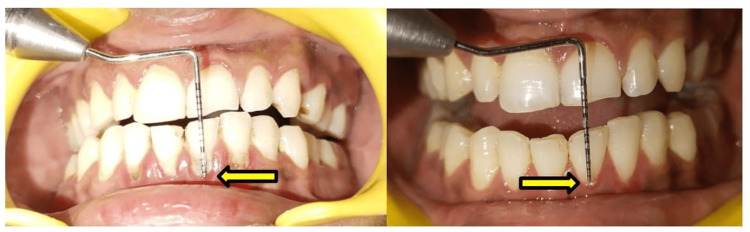
Pre-scaling and root planning (SRP) and post-SRP comparison

Another pre-operative and post-operative SRP case is shown in (Figure 4). We can appreciate the shift in the gingival margin after SRP (phase I therapy).

Statistical analysis

The IBM SPSS Statistics for Windows, Version 19 (Released 2010; IBM Corp., Armonk, New York) software was used for statistical analysis. A post hoc (Tukey) ANOVA test was used to compare measurements amongst diverse intra-group time frames.

## Discussion

Root coverage is a complex procedure influenced by numerous factors that ultimately determine the outcome. A plethora of literature highlights numerous surgical protocols for treating gingival recession. The term "creeping attachment" has already been mentioned in the past by several authors following surgical therapy. Previous studies have also indicated that creeping attachment is different from surgical attachment (bridging) and should be separated from each other. However, the difference in formation and pattern remains unclear. Moreover, the self-healing ability of the gingiva in terms of the genotype memory of periodontal tissues has not been previously discussed [1-6]. This study aimed to highlight the non-surgical management of the gingival margin position through creeping attachment in cases of Miller’s class I gingival recession.

Creeping attachment is a passive mechanism for covering the roots. In this study, a statistically significant improvement was observed in the marginal gingival position from baseline to 10 and 21 days after basic therapy. Matter and Cimasoni [10] have discussed creeping attachment as observed after gingival soft tissue grafting. They theorize that, firstly, creeping attachment supervenes as fibroblasts have the ability to contract, and it follows intracellular microfilament formation that contributes to the smooth muscular fibre characteristics of these cells. Secondly, creeping attachment might be due to over-healing; the grafted tissue has a tendency to establish induced newly formed attached gingiva and effectively cover the exposed root area by cell proliferation rather than by movement. Creeping attachment is also dependent on multiple factors, such as inter-proximal alveolar bone, gingival recession width, oral hygiene, and tooth and graft position [8,10]. Our study observed that the thickness of the gingiva undoubtedly plays an important role. Bell et al. [2] mentioned that creeping attachment was commonly seen in the lower anterior region during free gingival auto-graft healing. This attachment was characteristically formed from one month to a year. The post-operative coronal immigration recorded was 0.38-1.61 mm over a year. Pollack [11] reported bilateral creeping attachment in the maxillary canine region after autogenous gingival grafting. Parra and Capri [12] detected creeping attachment in peri-implant plastic surgical procedures. Perelli et al. [13] described a non-surgical method to advance gingival recession and thickness, and it was termed the "creeping attachment induced technique" (CAIT). The technique was instigated by de-epithelizing the boundaries of the gingival sulcus using a diamond bur, followed by the natural self-healing of soft tissues. The undistinguishable procedure was executed after four weeks, and a substantial amount of amplified gingival thickness and creeping attachment was noted within six months.

Soldatos et al. [14] observed creeping attachment formation within two months of treating gingival recession in individuals of the same gender and age. Meanwhile, Gul et al. [15], in a study, inferred that recession depth is a significant factor in determining the outcome of creeping attachment. Recently, a case report noted gingival creeping attachment after Miller’s class I gingival recession treatment. The technique used was vestibular incision subperiosteal tunnelling access, in combination with a connective tissue graft. Following mucogingival surgery, the process of gingival healing instigates a blood clot and granulation tissue formation with the thickening of the gingiva. This was followed by a maturation phase characterized by creeping attachment [16-18]. Creeping attachment is commonly believed to occur after mucogingival surgery. However, the outcome is uncertain, and the extent is unpredictable. Matter [19] reported a creeping attachment of 0.89 mm in the first year following free gingival graft surgery. Similarly, Nelson [20] reported 1-2 mm of creeping attachment formation 12 months after sub-pedicle connective tissue grafting. In addition, Fowler and Breault discussed that 1.0 mm of creeping attachment was observed after one month and was protuberant in just 10 days after a scalpel frenectomy procedure in the anterior mandible. Similar results were also given by Fowler and Breault [21] and Stylianou et al. [22]. In all the available literature, however, none of the case reports discussed the effects of SRP on the gingival margin position.

To the best of our knowledge, this is the first study explicitly designed to evaluate the effects of non-surgical periodontal therapy (such as SRP) on the creeping attachment of the gingiva. The results of the study showed that creeping attachment occurred as early as 10 days after basic treatment in cases of Miller class I gingival recession. After basic therapy, the inflammation subsided, leading to a firm and irrepressible consistency of the gingiva. Subsequently, the gingiva gradually attempted to regain its original position. It was also observed that adequate gingival thickness, absenteeism of plaque, and gingival inflammation are fundamental to achieving creeping attachment.

Strengths of the study

Novel Research

The leading strength of our study is its potential to serve as a template study for further research in this field. Due to the lack of available research, this genuine attempt to evaluate and quantify the creeping attachment before and after phase I periodontal therapy might be considered a significant contribution to addressing the gap in this area of periodontics.

Clinical Applicability

The study can strengthen clinical diagnosis and patient management in cases of class I gingival recession with adequate width. According to our study, these patients are the ideal candidates for developing creeping attachment after undergoing SRP alone, so major surgical invasion can be avoided, leading to better patient compliance.

Simple and Cost-Effective Study

The study is simple, cost-effective, and standardized in design. The findings were recorded by a single assigned observant to reduce measurement bias and maintain the standardization protocol.

Limitations of the study

Outcomes Cannot Be Weighed With Previous Research

The first notable limitation is the comparison with gold standards on this subject, as this study is the foremost research evaluating the gingival margin through creeping attachment before and after phase I periodontal therapy. Several previous studies have evaluated creeping attachment with mucogingival surgeries, but no studies have compared and investigated creeping attachment with SRP alone. Hence, the literature must be more detailed in order to compare the results with previous data.

Small Sample Size and Duration of the Study

While the study is adequate, a larger sample size and longer duration could have added more weight to this research. Therefore, it is recommended that additional longitudinal studies be conducted to complement this study.

## Conclusions

Creeping attachment is a passive attachment procedure in which the gingival margin shifts in a coronal way to cover the exposed root surfaces. Many researchers still view the biological explanation from a speculative perspective. Currently, creeping attachment formation around the natural teeth and dental implant is not copiously comprehended. Due to its inconsistent occurrence, creeping attachment cannot be considered an indisputable treatment modality for root coverage. However, it can be viewed as a non-surgical tact to preclude surgical intercession in a few cases.
